# Genome engineering of induced pluripotent stem cells to manufacture natural killer cell therapies

**DOI:** 10.1186/s13287-020-01741-4

**Published:** 2020-06-16

**Authors:** Keerthana Shankar, Christian M. Capitini, Krishanu Saha

**Affiliations:** 1 Department of Biomedical Engineering, University of Wisconsin-Madison, Madison, WI USA; 2 Department of Pediatrics, University of Wisconsin School of Medicine and Public Health, 1111 Highland Ave, WIMR 4137, Madison, WI 53705 USA; 3 University of Wisconsin Carbone Cancer Center, University of Wisconsin-Madison, Madison, WI USA; 4 Wisconsin Institute for Discovery, University of Wisconsin-Madison, 330 N Orchard St, WID 4164, Madison, WI 53715 USA

**Keywords:** Human induced pluripotent stem cells, Somatic cell gene editing, CRISPR-Cas9, Cancer, Immunotherapy, Cell therapy, Biomanufacturing, NK cells

## Abstract

Natural killer (NK) cells play a crucial role in host immunity by detecting cells that downregulate MHC class I presentation and upregulate stress ligands, as commonly seen in cancers. Current NK therapies using primary NK cells are prone to manufacturing issues related to expansion and storage. Alternative cell sources utilizing immortalized NK cell lines require irradiation and are dependent on systemic IL-2 administration, which has been associated with adverse effects. In contrast, NK cells differentiated from induced pluripotent stem cells (iPSC-NK cells) offer an off-the-shelf alternative that may overcome these bottlenecks. The development of a serum-free and feeder-free differentiation protocol allows for the manufacturing of clinically adaptable iPSC-NK cells that are equally as effective as primary NK cells and the NK-92 cell line for many indications. Moreover, genetic modifications targeting NK-mediated antibody-dependent cellular cytotoxicity capabilities, cytotoxicity, and checkpoint inhibitors may increase the therapeutic potential of iPSC-NK products. This review will highlight the current sources for NK therapies and their respective constraints, discuss recent developments in the manufacturing and genetic engineering of iPSC-NK cells, and provide an overview of ongoing clinical trials using NK cells.

## Introduction

Despite recent advances in standard of care, cancers still account for more than 600,000 deaths annually in the USA alone [1], causing researchers to investigate alternative therapies. Immunotherapy, which seeks to harness and augment natural functions of the patient’s immune system to treat tumors, has now generated numerous approaches: antibodies, cytokines, dendritic cell vaccines, checkpoint inhibitors, adoptive cell transfer with tumor infiltrating lymphocytes (TIL) or genetically modified T cells, or some combination of these treatments [2]. While many of these are promising options (reviewed elsewhere [2–4]), this review will focus on natural killer (NK) cell-based approaches and their potential for off-the-shelf therapeutics.

Progress with T cell therapies over the last decade has provided guidance for the development of NK cell therapies. One approach to T cell therapies is to use autologous or human leukocyte antigen (HLA)-matched allogeneic T cells with an engineered chimeric antigen receptor (CAR). These cells are genetically modified to express antibody fragments fused to T cell signaling domains, providing antigen-specific cytotoxicity independent of the HLA [5].

There are currently two Food and Drug Administration (FDA)-approved CAR T cell therapies, tisagenlecleucel [6–8] and axicabtagene ciloleucel [9, 10]. Both therapies employ a virally inserted CD19 CAR that allows T cells to recognize and kill malignant B cells expressing the CD19 antigen, including B cell acute lymphoblastic leukemia and non-Hodgkin lymphomas [6–10]. Patients undergoing CAR T cell therapies can experience remission of their cancer after receiving lymphodepletion chemotherapy followed by CAR T cell infusion, but can also be susceptible to on-target/off-tumor B cell aplasia, due in part to the formation of memory CAR T cells [6–10]. Patients may also develop cytokine release syndrome (CRS) and/or immune effector cell-associated neurotoxicity syndrome (ICANS), which require further treatment in an intensive care unit (ICU) due to high likelihood of progression into other life-threatening complications [6–10]. Although more effective than traditional chemotherapy, T cell therapies require long production times, are expensive (~$373–475,000 USD per dose) [11], and are also partly dependent on the quality of the leukapheresis product from the patient, making them harder to implement in large-scale treatments [12]. Allogeneic T cell therapies are also prone to these issues, in theory, and also require HLA matching or knockout of the T cell receptor (TCR) to prevent graft-versus-host disease (GvHD) [5, 13].

NK cells are another important component of the immune system which have proven to be effective at killing malignant cells [14–17]. In contrast to T cells, NK cells do not require antigen priming and are HLA agnostic—thus avoiding GvHD—making them an excellent candidate for off-the-shelf therapies [17–19]. Clinical trials using NK cells have been available for the last 20 years and currently involve treatment for hematologic malignancies and solid tumors (see Supplementary Table 1, Additional file 1) [19–27]. Most of these trials examine the maximum tolerated dose of ex vivo activated and expanded primary peripheral blood NK cells (PB-NK), primary cord blood NK cells (CB-NK), or immortalized NK cell lines (e.g., NK-92 cells), while some trials focus on genetically modified NK cells, including CAR NK cells. Several trials using PB-NK cells implement lymphodepleting chemotherapy, and some trials administer the NK cells in combination with interleukin-2 (IL-2) or FDA-approved monoclonal antibodies to promote antibody-dependent cellular cytotoxicity (ADCC). However, the use of autologous and allogeneic NK cells in nearly all of these trials leads to the same long production times of weeks as observed with T cells. Developing NK cell-based immunotherapies from induced pluripotent stem cells (iPSCs) circumvents these issues while providing off-the-shelf capabilities. Additionally, genetic modifications—similar to those in T cells—can further improve the specificity, strength, and efficacy of iPSC-NK cell therapies. In this review, we will discuss the current sources for NK cells and recent developments in gene-modified iPSC-NK cells for universal therapies applicable to a global population.

## Activation and inhibition of NK cells

NK cells are large granular lymphocytes present in the innate immune system that provide defense against virally infected cells and malignant tumors. In humans, NK cells are often defined by the presence of CD56, a marker also detected on other lymphocytes including CD8^+^ T cells, dendritic cells, and γδ T cells, and by their lack of CD3 [28–31]. NK cells also express the low-affinity Fcγ receptor CD16 [32, 33] to mediate ADCC and can generally be classified into two major subsets: CD56^bright^CD16^+^ and CD56^dim^CD16^+^ [31]. Although there is evidence of a small population of CD56^neg^CD16^+^ NK cells in healthy individuals that undergoes expansion during HIV-1 infection [34], the CD56^bright^ cells are abundant in lymphoid and nonlymphoid tissues and exhibit high cytokine secretion [35–37]. CD56^dim^ cells are prevalent in peripheral blood and exhibit high cytotoxicity through secretion of perforin and granzyme B to kill target cells [35].

**Fig. 1:**
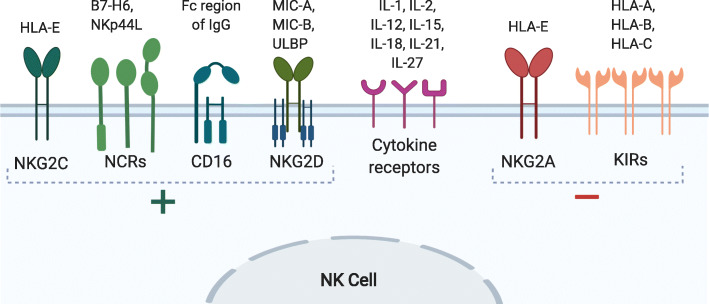
Major NK cell receptors and ligands. NK cells express a repertoire of activating (“+”; NKG2C, NCRs, CD16, NKG2D) and inhibitory (“−”; NKG2A, KIRs) receptors on their cell surface. The activation of NK cells is dependent on the integration of the signals received by these receptors. The ligands responsible for NK activation and inhibition are shown above their corresponding receptors. Additionally, NK cells also express cytokine receptors that regulate cell functions such as proliferation and persistence and, in certain disease contexts, a memory-like phenotype

**Fig. 2:**
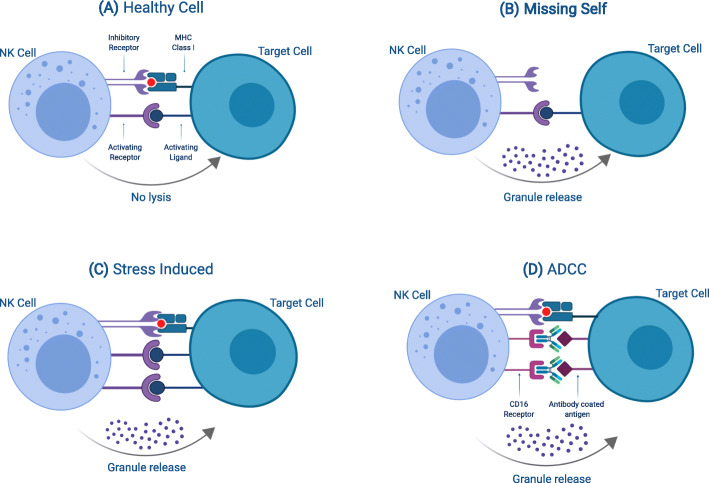
Schematic of NK cell functions. **a** In normal cells, MHC class I molecules can engage inhibitory receptors on NK cells that can outnumber and override activating receptors, preventing NK-mediated cytotoxicity. **b** In malignant cells, downregulation of MHC class I molecules can result in the absence of NK inhibitor receptor engagement, resulting in NK-mediated cytotoxicity. **c** In cases where tumor cells retain expression of MHC class I molecules, NK cytotoxicity can still be induced through the upregulation of stress-induced activation ligands that can bind activation receptors on NK cells. **d** In instances when target cells are opsonized by antibodies, NK cells can exhibit ADCC by detecting antibodies on the target cell through the low-affinity Fcγ CD16 receptor. The outcome of the NK cell response is determined by the amount of activating versus inhibitory receptors on the NK cell surface

NK cell activation is dependent on the integration of signals received by the multitude of membrane bound activating and inhibiting receptors (Fig. 1). This unique regulation allows NK cells to mount an immune response that is independent of antigen presentation. NK cells distinguish healthy cells from abnormal cells through detection of “self-ligands” such as major histocompatibility complex (MHC) molecules [38]. The detection of MHC molecules from the MHC class I recognizing receptors, such as killer cell immunoglobulin-like receptors (KIR) and lectin-like NKG2A heterodimers, results in inhibitory signals (Fig. 2a) [38]. Malignant tumors have been shown to downregulate MHC molecules [39, 40] and upregulate stress ligands such as MICA and MICB [41–43], rending them susceptible to NK lysis (Fig. 2c). In cases when malignant cells escape immune surveillance through normal MHC expression, NK cytotoxicity can still be induced through sufficient stimulation of activating receptors (Fig. 2b), including the stress ligand receptor NKG2D [44], and natural cytotoxic receptors (NCR) NKp30 [45], NKp44 [46], and NKp46 [47]. In instances when target cells are opsonized with antibodies, the CD16 receptor on NK cells can bind to the Fc portion of the immunoglobulin G (IgG) and trigger granule secretion (Fig. 2d). Though NK cells are part of the innate immune response, there is evidence indicating that they are capable of forming a memory-like phenotype in acute myeloid leukemia (AML) [48]. Thus, NK cells provide additional levels of immune surveillance by targeting tumors that evade T cell-mediated killing.

## Current sources of NK cells

**Fig. 3:**
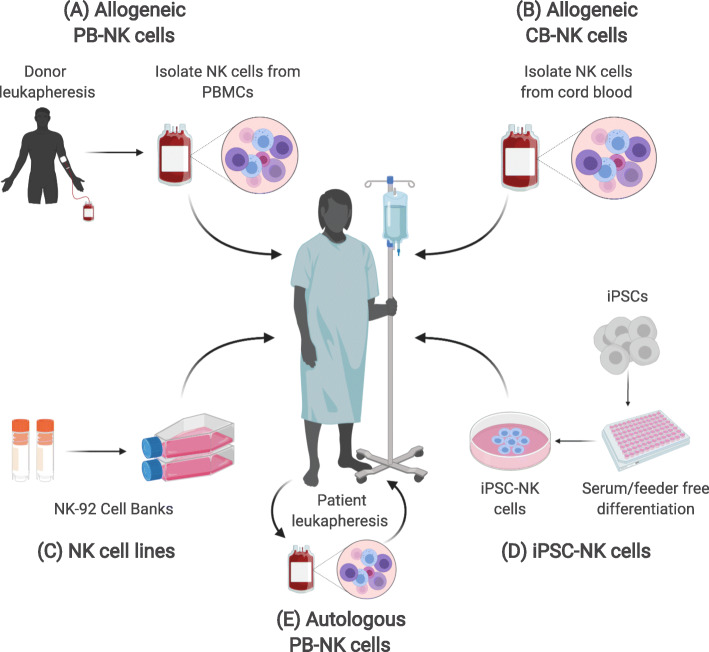
Sources of NK cells. Current NK therapies use NK cells from the following sources: primary PB-NK cells, primary CB-NK cells, NK cell lines, and recently, iPSC-NK cells, each with its own benefits and limitations. See Supplemental Tables 1 and 2 (Additional files 1 and 2) for a list of products in clinical trials

Current immunotherapies employ NK cells from various sources: primary PB-NK cells (Fig. 3a, e), primary CB-NK cells (Fig. 3b), immortalized NK cell lines (Fig. 3c), and recently, iPSC-derived NK cells (Fig. 3d), each with its own benefits and disadvantages. Studies in the late 1990s to early 2000s focused on improving anti-tumor activity of NK cells using cytokine activated, autologous primary NK cells [49–51]. Isolating large numbers of primary PB-NK cells, however, has proven to be difficult as only 5–15% of circulating blood lymphocytes are NK cells, and quantities derived from leukapheresis products are highly donor dependent [52–54]. Additionally, isolation from peripheral blood lymphocytes often results in a heterogenous population of lymphocytes with only a 10–20% yield of NK cells [55]. The use of primary NK cells is further complicated by their decrease in cytotoxicity following cryopreservation [56, 57]. While the low yield and loss in cytotoxicity from cryopreservation can be partially overcome with cytokine support and feeder cell expansion, this approach makes PB-NK cells suboptimal for an “off-the-shelf” therapy [58–60]. In contrast, CB-NK cells recover well after cryopreservation and may be a potential candidate for “off-the-shelf” therapy using primary NK cells [61]. Though there is a greater percentage of NK cells in CB (23%) than in PB (11%), one disadvantage is the limited number of NK cells per cord blood unit, potentially requiring expansion of multiple units per dose [62]. When compared to PB-NK cells, CB-NK cells express lower levels of KIRs and granzyme B and higher levels of the NKG2A inhibitory receptor, suggesting that CB-NK cells have an immature phenotype [62, 63]. While this particular phenotype results in less potent cytotoxicity, CB-NK cell effector function can be improved to that of PB-NK cells with cytokine stimulation [63].

Alternative approaches have been considered using immortalized NK cell lines. Eight malignant NK cell lines have been established: NK-92, YT, NKL, HANK-1, KHYG-1, NK-YS, NKG, and NK101 [64, 65]. Of the eight cell lines, NK-92 is the only established cell line that is FDA approved for patient testing in phase 1 and 2 clinical trials [27, 66–69]. The NK-92 cell line was established from the blood of 50-year-old male patient with rapidly progressive non-Hodgkin lymphoma [70]. The NK-92 cell line can easily be maintained in vitro and expanded to large numbers through good manufacturing practices (GMP) with the addition of recombinant IL-2 [71]. Unlike primary NK cells, the NK-92 cell line can be cryopreserved with high recovery and function post-thaw [72]. NK-92 cells lack most of the KIRs while expressing several activating receptors making them suitable for anti-tumor therapies [73]. However, one disadvantage of NK-92 cells is that they do not express the low-affinity Fcγ CD16 receptor, making them incapable of an ADCC response [70]. To address this, NantKwest has developed an NK product engineered with a high-affinity CD16 receptor using their proprietary NK-92 platform [74]. The product, haNK, has been tested in a phase I clinical trial for solid tumors and is undergoing phase II clinical trials for Merkel cell carcinoma (see Supplementary Table 1, Additional file 1, ClinicalTrials.gov: NCT03027128, NCT03853317). Additionally, the malignant origin of the cell line requires them to be irradiated before adoptive transfer into patients [27, 66–69], inhibiting their proliferative capacity and persistence in vivo from a range of 48 h to 1 week, depending on modifications such as CARs [68, 69]. It has been shown previously using PB-NK cells that increased in vivo persistence (> 7 days) correlates with greater anti-tumor activity [75], weakening the rationale for using NK-92 therapies unless serial dosing is utilized. While multi-dose treatments would circumvent this issue, it would also require systemic administration of IL-2 to support NK growth, which may trigger additional side effects such as vascular leak syndrome and upregulation of regulatory T cells, which suppress NK cells [76–78].

Recently, there has been increasing interest in iPSC-NK therapies due to their ability to address the supply-chain bottlenecks associated with primary and cell line NK therapies. Advantages include that iPSCs can be generated from easily accessible sources such as fibroblasts or peripheral blood, retain pluripotency during expansion, and be banked for long-term storage [79]. NK cells derived from iPSCs have proven to be equally as effective as primary NK cells and NK-92 cells. One study showed that iPSC-NK cytotoxicity is comparable to activated and expanded PB-NK in MA148 and A1847 ovarian tumor xenograft models [80]. Donor peripheral blood-iPSC-NK cells have also been shown to have greater cytotoxicity against SKOV3, SW480, HCT-8, MCF7, and SCC-25 cancer cell lines compared to donor PB-NK cells, and similar efficacy against K562s as donor PB-NK cells [81]. iPSC-NK cells also prove advantageous over the established NK-92 cell line in that iPSC-NK cells do not need to be irradiated [80–82]. Another benefit of iPSC-NK cells is that they express the CD16 receptor and are therefore capable of ADCC [81, 82]. Zeng et al. demonstrated that PB-iPSC-NK cells are capable of ADCC by showing successful killing of Raji cells opsonized with an anti-CD20-hIgG1 antibody [81]. Typical degranulation of NK cells is achieved through phosphorylation of the Syk-PLCγ-DAG/inositol triphosphate or Syk-Vav-RAC-PAK-MEK-ERK pathways, while the secretion of inflammatory cytokines results from phosphorylation of the NF-κB pathway [83, 84]. However, it is not yet entirely clear if iPSC-NK cells require the same activation pathways to induce granule and cytokine secretion. Future studies need to mechanistically interrogate the signaling pathways activated upon iPSC-NK stimulation. Still, these data suggest that iPSC-NK-based strategies combine the most attractive qualities of primary NK cells and the NK-92 cell line, namely high potential for cytotoxicity, including ADCC function, and potential for expansion and persistence in vivo, even after cryopreservation.

## Manufacturing NK cells from iPSCs

**Fig. 4:**
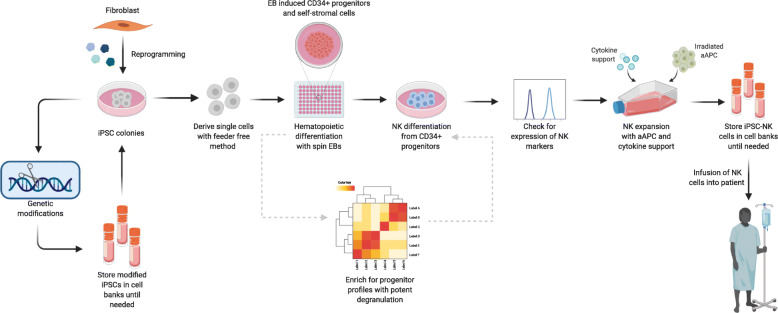
A potential pipeline to manufacture iPSC-NK cells. At the iPSC stage, the cells can be modified and cryopreserved as a master cell bank for later use, or directly differentiated into NK cells. Spin-embryoid bodies can be used to induce CD34^+^ progenitors and self-supporting stromal cells without the use of feeder cells. The CD34^+^ progenitors can be differentiated into NK cells before expansion with cytokines and irradiated artificial antigen presenting cells (aAPCs). At this stage, the iPSC-NK cells can be cryopreserved as a working cell bank until infused into the patient. It may also be useful to enrich the CD34^+^ progenitor population (indicated by gray arrows) for cells with potent degranulation profiles before differentiating into NK cells

Serum-/feeder-free iPSC-NK derivation methods have already been established, in contrast to iPSC-T cell differentiations, which largely require xenogeneic Notch signaling from feeder cells [85–87]. A serum-, stromal, and feeder-free method for differentiation has recently been shown to repeatably produce clinical-scale mature NK cells in just 5 weeks (Fig. 4) [88] as opposed to a previously published longer protocol [89]. This new method eliminates the need for feeder-dependent single-cell adaptation, a non-uniform process that typically takes 2–3 months, and updates it to include mTeSR and Matrigel for quicker and more controlled single-cell iPSC generation [88].

Another advantage to using iPSC-NK products, compared to iPSC-T cell therapies, is that iPSC-NK products have the capacity to be truly off-the-shelf as they would not need to be HLA matched between donors and patients. To create a universal therapy using iPSC-T products, cell banks would still require several different HLA diverse iPSC lines to cater to a large population. Okita et al. found that 50 unique HLA iPSC lines are required to treat over 73% of the Japanese population [90]. Taylor et al. found that 150 homozygous HLA iPSC lines are required to match 93% of the UK population [91]. One strategy to overcome the high number of required cell lines regarding iPSC-T therapies would be to genetically delete the HLA genes, but this particular modification is not necessary in iPSC-NK cells. However, the biomanufacturing time of 5 weeks for iPSC-NK cells could be a limitation to establishing and maintaining iPSC-NK cell banks, especially if genetic modifications such as CARs are necessary. Strategies to shorten and streamline the manufacturing process would prove useful.

Groups have successfully differentiated pluripotent stem cells (PSCs) into NK cells that are similar in phenotype and effector function when compared to primary NK cells [80, 81, 89, 92–98]. The differentiated cells express several NK-associated receptors such as CD56, KIRs, CD16, NKp44, NKp46, NKG2D, and TRAIL [80, 82, 92–95]. However, it is unclear if the derived cells resemble the bright or dim subset of the CD56 population, suggesting that further investigation into the expression profile of differentiated NK cells may be necessary. A recent study by Dege et al. further probed the ontogeny of hPSC-derived NK cells and found two distinct CD34^+^ populations that dictate final effector function [97]. The HOXA^neg/low^/CD34^+^ progenitors gave rise to a population that exhibits more potent degranulation while the HOXA^+^/CD34^+^ progenitors resulted in an NK population with robust inflammatory cytokine secretion [97]. These findings suggest that the origin of differentiated NK cells should be considered when adapting for immunotherapy applications and that it may be beneficial to enrich progenitor populations for markers of a more potent degranulation profile during differentiation.

Given the therapeutic potential of iPSC-NK products, the FDA has already approved a phase I clinical trial to investigate Fate Therapeutics’ off-the-shelf iPSC-NK product, FT500 [99]. FT500 is the first FDA-approved clinical investigation of an iPSC-derived cell product in the USA [99]. The NK cells are developed from a clonal master iPSC line cell bank, therefore making it possible to mass produce iPSC-NK cells that are relatively homogenous, quality controlled, and able to be cryopreserved for long-term storage [100]. The trial seeks to assess the safety and tolerability of multiple doses of FT500 in combination with checkpoint blockade therapy to treat adults with advanced solid tumors (see Supplementary Table 2, Additional file 2, ClinicalTrials.gov: NCT03841110). A separate observational study has been initiated to assess the long-term safety and efficacy of patients who receive FT500 treatment (see Supplementary Table 2, Additional file 2, ClinicalTrials.gov: NCT04106167).

## Genetic modification of iPSC-NK cells

**Fig. 5:**
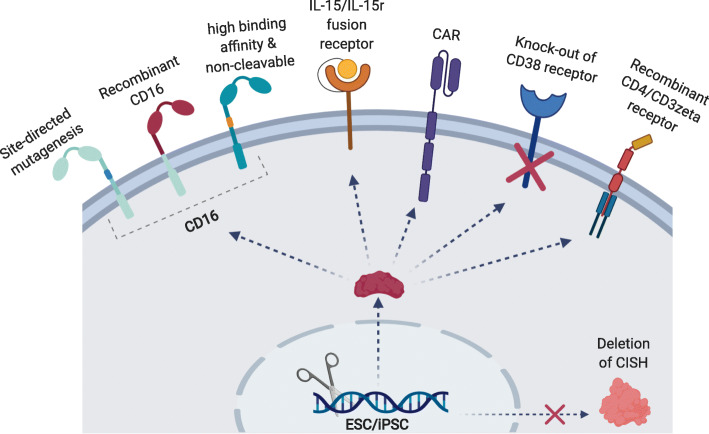
A summary of genetically modified iPSC-NK therapies. iPSC-NK cells have been genetically engineered to overcome the various limitations associated with NK therapies. Attempted modifications include mutated versions of the CD16 receptor to inhibit cleavage by the ADAM17 protease, fused cytokine receptor-ligand receptors to improve in vivo persistence, CARs to improve anti-tumor cytotoxicity, receptor knockout to prevent fratricide from combinatorial antibody treatments, and recombinant receptors to increase NK cytotoxicity outside the context of cancer. Another approach has been to target checkpoint inhibitors through deletion of the *CISH* gene

Despite the promising functionality of NK cells for treating cancer, the cells can become less effective over time due to a decrease in NK persistence and cytotoxicity. Therefore, genetic modifications to overcome these issues would prove useful to enhance antigen specificity and cytotoxicity (Fig. 5). Though groups have successfully modified primary NK cells and NK-92 cells using retroviral [101–106], lentiviral [107–113], and mRNA transfection [107, 114–117] approaches, there is wide variability in the efficiencies of transduction/transfection across these studies. The range of efficiencies span from 13 to 69% for retroviral transductions [101–106], 4 to 90% for lentiviral [107–111], and < 10 to > 60% for mRNA transfections [107, 114–117]. It is feasible to modify iPSCs using similar approaches including viral vectors, transposons, and CRISPR-Cas9 [85, 118–124]. Moreover, editing at the pluripotent stage would result in fully edited clonal lines that can be differentiated towards a NK lineage.

One of the issues associated with loss of cytotoxicity is the cleavage and shedding of the CD16 receptor. Upon NK activation, CD16 undergoes cleavage by the ADAM17 protease and is shed from the membrane, causing the NK cell to lose its ability to perform ADCC [121]. To circumvent this issue, Jing et al. transduced a mutated version of the CD16a receptor (CD16a/S197P) into iPSCs using a *sleeping beauty* transposon [121]. A site-directed mutagenesis of the CD16a receptor (S197P) prevented cleavage by ADAM17 and resulted in stable expression of CD16 even upon activation by the K562 cell line. In a separate abstract, Blum et al. reported that the CD16a/S197P-transduced iPSC-NK cells were 97.5% CD16^+^ before stimulation and 95.2% CD16^+^ after stimulation [122]. They also reported that CD16a/S197P-transduced iPSC-NK also showed improved degranulation and better killing of the SKOV3 ovarian cancer cell line when compared to unmodified iPSC-NK and PB-NK [122]. Studies incorporating CRISPR/Cas9 are also underway to determine the larger effects of deleting the ADAM17 gene [122]. Another successful modification to prevent CD16 cleavage has been to transduce iPSCs with a recombinant Fcγ receptor with the extracellular region of CD64, and the intracellular and transmembrane region of CD16a (CD64/16A) using a *sleeping beauty* transposon [124]. The CD64/16A receptor lacks the ADAM17 cleavage site, preventing CD16 downregulation upon NK activation. In an in vitro cytotoxicity assay, the transduced iPSC-NK cells exhibited greater ADCC against SKOV3 ovarian cancer cells when compared to untransduced iPSC-NK cells.

Fate Therapeutics is conducting an analogous line of research with their iPSC-NK products, FT516 and FT538 (see Supplementary Table 2, Additional file 2). FT516 is an iPSC-NK product that has been engineered with a high-affinity, non-cleavable CD16 (hnCD16) receptor at the iPSC stage to enhance ADCC and anti-tumor capabilities, and is undergoing phase I clinical trials in adults with hematologic malignancies (see Supplementary Table 2, Additional file 2, ClinicalTrials.gov: NCT04023071) [125]. A preclinical study reported that hnCD16-iPSC-NK cells displayed greater ADCC capabilities, CD107a expression, and IFN-gamma production compared to peripheral and unmodified iPSC-NK cells against various antibody-treated cancer cell lines [126]. While treatment with hnCD16-iPSC-NK, iPSC-NK, or PB-NK cells alone did not show a change in tumor burden, a combinatorial treatment of anti-CD20 and hnCD16-iPSC-NK showed a decrease in tumor burden 10 days after treatment in an in vivo B cell lymphoma mouse xenograft model. However, relapse occurred in most treated groups, but was rescued by multiple doses of treatment extending the mean survival from 52 to 76 days. The FT538 product also addresses issues with NK cytotoxicity, specifically in cases of multiple myeloma. Myeloma cells strongly express CD38 and are often treated with daratumumab, an anti-CD38 monoclonal antibody [127]. However, administration with daratumumab has been shown to demonstrate reductions in NK cell counts and activation due to fratricide from NK expression of CD38 [128]. To circumvent this, FT538, derived from a clonal master iPSC line, has been modified with a knockout of the CD38 receptor and knock-in of the hnCD16 receptor to increase ADCC and prevent exhaustion when used with anti-CD38 antibody treatments [129].

Though useful strategies, completely preventing cleavage of CD16, could also be problematic since CD16 shedding has been established as a regulatory mechanism that sustains NK survival by assisting with detachment of NK cells from target cells [130]. Once a target cell is lysed, the NK cell detaches and continues its surveillance for other malignant cells and can normally kill up to seven target cells in a 12-h period [131]. However, Srpan et al. observed a 67% decrease in detachment of NK cells and found that NK cells stayed in contact with their target cells even after 8 h when modified with the mutant CD16 receptor (S197P) from the Jing et al. study [130]. While the cytotoxicity of the NK cells was not affected, there was less target cell lysis overall. The authors also noted an increase in NK cell death from 23 to 38% in the presence of an ADAM17 inhibitor due to the inability of the NK cell to detach itself [130]. These results suggest that a fully non-cleavable CD16 receptor could be advantageous initially but may be a limiting factor for “serial killing.” Moreover, constant engagement of the NK cells may also lead to a hyperinflammatory environment due to increased cytokine secretion, causing deleterious side effects such as CRS that could contribute to morbidity or mortality. Strategies to induce CD16 re-expression or increase the rate of CD16 receptor turnover after shedding may provide more effective alternatives.

iPSC-NK cells have also been investigated in the context of human immunodeficiency virus/acquired immunodeficiency syndrome (HIV/AIDS) due to the ability of NK cells to kill virally infected cells. Ni et al. found that iPSC-NK cells were capable of inhibiting replication of HIV-infected CD4^+^ T cells through degranulation, cytokine secretion, and ADCC within in vitro co-culture assays [94]. In another study, Ni et al. engineered embryonic stem cells (ESCs) and iPSCs with a receptor composed of the extracellular region of CD4 and the intracellular signaling chain of CD3ζ before differentiating to NK cells to target HIV-infected cells [98]. The engineered ESC/iPSC-NK cells showed better suppression of HIV-infected T cells in in vitro assays when compared to unmodified NK cells. In a humanized mouse model, the authors found inhibition of HIV replication in mice treated with modified and unmodified ESC/iPSC-NK cells, but noted that the difference was not significant between the two groups. These findings suggest that iPSC-NK therapies, modified or not, may provide potential treatments outside the context of cancer.

## CAR NK and iPSC-CAR-NK cells

Another proposed approach to increasing the anti-tumor activity of NK cells has been to engineer them with CARs. Clinical trials have been initiated for CAR NK treatments using donor derived primary NK cells (see Supplementary Table 1, Additional file 1, ClinicalTrials.gov: NCT03056339, NCT00995137) and completed but not yet published (NCT00995137). A phase I/II clinical trial administering cord blood CAR NK cells for relapsed or refractory CD19-positive cancers showed encouraging outcomes with seven of eleven patients experiencing complete remission, with no development of CRS or ICANS noted (ClinicalTrials.gov: NCT03056339) [132]. The CAR NK treatment, coined “TAK-007,” is currently being developed by Takeda Pharmaceuticals, alongside three other CAR NK treatments, in anticipation of a multicenter phase II clinical trial in 2021 [133]. Other immunotherapy companies, such as Nantkwest and Nkarta, are also conducting preclinical research for CAR NK products for B cell malignancies and solid tumors. NantKwest is developing a CAR NK product (taNK) using its uniquely engineered NK-92 platform for HER2/NEU expressing cancers [74]. Nkarta Therapeutics is working to create NKX101, a unique CAR NK therapy that targets NKG2D stress ligands in place of cancer-specific antigens, and an anti-CD19 CAR NK product [134].

Given the accessibility of iPSCs [79] and efficacy of iPSC-NK cells [80–82], it can be beneficial to engineer CARs in iPSCs. For instance, the Kaufman group has had success in engineering iPSCs with an optimized NK-CAR construct constructed with an anti-mesothelin scFv region, NKG2D transmembrane domain, 2B4 co-stimulatory domain, and a CD3ζ cytotoxicity domain using a *PiggyBac* transposon vector, prior to differentiation into functional CAR-iPSC-NK cells [120]. The derivation efficiency of NK cells from CAR bearing iPSCs was similar to the efficiency from unmodified iPSCs, and the phenotype of the CAR-iPSC-NK cells was comparable to that of the PB-NK cells. In an ovarian cancer xenograft model, NK-CAR-iPSC-NK cells displayed better anti-tumor activity when compared to PB-NK, iPSC-NK, and T-CAR-expressing-iPSC-NK cells and had greater circulation at day 10. When compared to third-generation CAR T cells, NK-CAR-iPSC-NK cells demonstrated similar anti-tumor effects but with less overall toxicity in vivo. Through a mechanistic study with site-specific mutations in each CAR signaling domain, the authors also demonstrated that the cytotoxicity of the NK-CAR-iPSC-NK cells resulted from activation of established NK signaling pathways, Syk-vav1-Erk and NF-κB. Despite the positive results, the NK-CAR-iPSC-NK cells had limited in vivo persistence as they returned to baseline levels similar to those of PB-NK cells by day 21 and were barely detectable at day 28. The Rezvani group reported that the inclusion of an IL-15 domain in an anti-CD19 CAR resulted in detection of cord blood CAR NK cells up to 68 days after infusion [106]. Though the microenvironment of solid tumors adds an extra layer of complexity, the Rezvani group’s CAR could provide a blueprint for improving the in vivo lifespan of CAR NK cells as in vivo persistence has been correlated with the efficacy in CAR T cell treatment for solid tumors [135].

Along a similar line of research, Fate Therapeutics has also been conducting preclinical investigations regarding their iPSC-NK product FT596 (see Supplementary Table 2, Additional file 2), which has been modified to include an anti-CD19 CAR, a hnCD16 receptor, and a novel IL-15/IL-15r fusion receptor to target B cell malignancies [136]. The CD19-CAR enhances anti-tumor activity against CD19 expressing B cells, while the CD16 receptor increases ADCC, and the novel IL-15/IL-15r fusion receptor promotes in vivo persistence and expansion. FT596 is being developed for use as a monotherapy or in combination with monoclonal antibodies. It has been shown to kill both CD19^+^ and CD19^−^ B cells in a co-culture assay when used in conjunction with rituximab, a monoclonal antibody for CD20 expressing B cells [136]. It has also been shown to prevent tumor progression and promote survival in a B cell leukemia xenograft model [136].

## Limitations of NK cell therapies

Despite the encouraging results of preclinical and clinical studies of NK therapies, there are still significant barriers to overcome with current approaches. Though efforts have been made to improve in vivo persistence through cytokine support, the short lifespan of NK cells in vivo may require multiple infusions. Investigations into serial dosing of cryopreserved stocks, combinatorial approaches with other immunotherapies such as CAR T cells, and using cytokines such as IL-15/IL-15r or IL-21 for additional support will be required.

An alternative approach to enhance NK effector functions has been to target checkpoint inhibitors of NK cells, such as the downregulation of IL-15 signaling. IL-15 is a critical chemokine involved in maintaining NK homeostasis [137]. However, it has been shown that the CIS regulatory element, encoded by the *CISH* gene, negatively regulates IL-15 signaling and thus acts as a major inhibitory checkpoint [138]. Using a murine model, Delconte et al. showed that CISH^−/−^ mice were immune to the formation of melanoma, breast, and prostate cancer in vivo due to an upregulation of IL-15 signaling in NK cells [138]. To overcome this inhibitory checkpoint, the Kaufman group used the CRISPR/Cas9 system to delete *CISH* at the iPSC stage prior to NK differentiation. When compared to unmodified iPSC-NK cells, the CISH^−/−^ iPSC-NK cells demonstrated better cytotoxicity against AML cell lines and greater in vivo persistence in an AML xenograft model [123]. Biallelic deletion of *CISH* also increased sensitivity to IL-15 and resulted in greater expansion when compared to unedited iPSC-NK cells, even at low concentrations of IL-15. In addition, groups are also examining the combination of adoptive NK cell infusions with checkpoint blockade antibodies such as anti-PD1, anti-TIM3, and anti-TIGIT to overcome NK exhaustion [139, 140], although there is some debate on whether PD-1 is a relevant checkpoint on NK cells [141]. Zhang et al. showed that blockade of TIGIT, an immunosuppressive factor, improved murine NK cell anti-tumor activity and prevented exhaustion in vivo of tumor bearing mice, suggesting that blocking inhibiting receptors may be a compelling strategy to improve anti-tumor responses [140].

As is the case with other immunotherapies [142], NK cells struggle to infiltrate solid tumors. Tumor penetration, in combination with tumor homing, has been strongly correlated with better prognosis [143, 144]. One study’s single-cell RNA-seq analysis showed that murine NK cells represented less than 1–5% of immune cells present in mouse tumor microenvironments, indicating that NK cells do not naturally infiltrate tumors at high percentages [145]. CXCR3 has been identified as a crucial receptor for NK chemotaxis in response to cancer-secreted ligands, CXCL9, CXCL10, and CXCL11 [146]. Therefore, engineering a CXCR3 receptor fused with NK activating signaling domains into an iPSC line may prove beneficial to enhance homing of NK cells to chemokine-secreting tumors. Even if NK cells penetrate the tumor, they encounter an array of immune suppressive factors, such as tumor secreted transforming growth factor-beta (TGF-β), that may downregulate NK activity regardless of genetic modifications [147–149]. It has been suggested that a dominant negative TGF-β receptor that inhibits TGF-β signaling or a chimeric receptor that converts TGF-β to an activation signal may provide potential strategies to overcome NK cell exhaustion [150, 151].

Although some of the studies investigating NK cell biology were performed in the context of murine NK cells, there is evidence that regulatory mechanisms may be conserved between human and murine NK cells. For instance, though the human KIR inhibitory receptor is structurally different from its murine analog, Ly49, both receptors contain an intracellular immunoreceptor tyrosine-based inhibitory motif (ITIM) that phosphorylates similar pathways to inhibit NK activation [152]. Additionally, the localization of receptors and ligands in chromosomes are similar in human and murine NK cells, suggesting that the regulatory mechanisms may be comparable in both populations [152]. However, murine NK cells lack CD56 and are identified through the expression of other markers such as NK1.1 (CD161b, CD161c), DX5 (CD49b), and NKp46 instead. Since the lack of CD56 expression on murine NK cells makes it difficult to identify analogous subsets in human NK populations, there is a possibility that the CD56^bright^ and CD56^dim^ subsets in humans may not have a comparable population in mice, and thus observations from comparable murine NK effector phenotypes may not translate to the clinic [153]. Nevertheless, these differences may be accounted for through appropriate manipulation of mouse models and understanding of their limitations.

## Concluding remarks

NK-based immunotherapies already provide a promising approach to treat several types of cancers (see Supplementary Table 1, Additional file 1). These therapies are likely complementary to T cell therapies and may be able to address some of the limitations of T cell therapies (e.g., requiring patient-donor HLA matching, development of CRS/ICANS, and the lack of a clinically adaptable differentiation protocol for iPSC-T products). Importantly, NK-mediated immunotherapy can be applied across all HLA haplotypes without the occurrence of GvHD. Moreover, iPSC technology offers new solutions for obstacles associated with conventional NK therapies regarding manufacturing, banking, and improving anti-tumor activity.

Given the advances made in the development and engineering of iPSC-NK products, it is essential to further examine the therapeutic potential of iPSC-NK immunotherapies in both preclinical and clinical studies, while trying to reduce biomanufacturing time. An “omics”-based approach to resolve tumor-NK cell interactions at the single-cell level may offer new targets to improve persistence in the tumor microenvironment. It would be of interest to examine the transcriptome and secretome of NK cells capable of infiltrating the tumor, fluctuations in NK gene expression induced from cancer cells, and changes, or lack thereof, in the tumor transcriptome in response to NK cell presence. We anticipate that an increase in our understanding of fundamental NK and tumor biology will soon prove useful in selecting appropriate gene modifications and further optimizing the biomanufacturing of NK cell immunotherapies.

## Supplementary information

**Additional file 1.** Selected Clinical Trials with Primary NK Cells and NK-92 Cells. The table provides an overview of selected clinical trials with normal and gene modified primary NK and NK-92 cells for hematological and solid tumors.

**Additional file 2.** Summary of iPSC-NK Cell Therapies. The table summarizes the iPSC-NK cell therapies reviewed in the manuscript.

## Data Availability

Not applicable.

## Abbreviations

aAPC: Artificial antigen presenting cells
ADCC: Antibody-dependent cellular cytotoxicity
AIDS: Acquired immunodeficiency syndrome
AML: Acute myeloid leukemia
CAR: Chimeric antigen receptor
CB-NK: Cord blood natural killer
CRS: Cytokine release syndrome
ESC: Embryonic stem cells
FDA: Food and Drug Administration
GMP: Good manufacturing practices
GvHD: Graft-versus-host disease
HIV: Human immunodeficiency virus
HLA: Human leukocyte antigen
ICU: Intensive care unit
IL-2: Interleukin-2
IL-15: Interleukin-15
IgG: Immunoglobulin G
iPSC: Induced pluripotent stem cells
ITIM: Immunoreceptor tyrosine-based inhibitory motif
KIR: Killer cell immunoglobulin-like receptor
MHC: Major histocompatibility complex
NCR: Natural cytotoxic receptor
NK: Natural killer
PB-NK: Peripheral blood natural killer
PSC: Pluripotent stem cells
TCR: T cell receptor
TGF-β: Transforming growth factor-beta
TIL: Tumor infiltrating lymphocytes
