# Supplementary material for: Genome engineering of induced pluripotent stem cells to manufacture natural killer cell therapies
Source: Stem Cell Res Ther. 2020 Jun 16;11:234. doi: 10.1186/s13287-020-01741-4 (PMC7298853; doi:10.1186/s13287-020-01741-4)
Supplement: Supplementary file 1 — Additional file 1. Selected Clinical Trials with Primary NK Cells and NK-92 Cells. The table provides an overview of selected clinical trials with normal and gene modified primary NK and NK-92 cells for hematological and solid tumors. [file 13287_2020_1741_MOESM1_ESM.pdf]

Supplementary Table 1. Selected Clinical Trials with Primary NK Cells and NK-92 cells (As of May 9, 2020)

Autologous NK Cells

Genome Modification(s)	Status	Phase	Trial Number	Condition	Manufacturing Notes	Treatment Notes	Trial Goals	Ref.
None	Recruiting	1	NCT00720785	Chronic myeloid leukemia, multiple myeloma, non-small cell lung carcinoma	NK cells are activated and expanded *ex vivo*	Preparative lymphodepletion regimen followed by infusion of escalating doses of NK cells after treatment with bortezomib	Determine safety of escalating doses of NK cells	-
None	Recruiting	1	NCT02573896	Neuroblastoma	NK cells are activated and expanded *ex vivo*	Preparative lymphodepletion regimen followed by infusion of NK cells and ch14.18 (GD2 antibody)	Determine feasibility of expanding, cryopreserving, shipping and infusing multiple doses of NK cells and determine maximum tolerated dose	-

Allogeneic NK Cells

Genome Modification(s)	Status	Phase	Trial Number	Condition	Manufacturing Notes	Treatment Notes	Trial Goals	Ref.
Anti-CD19 CAR (anti-CD19-BB-zeta)	Completed	1	NCT00995137	Relapsed or refractory B-lymphoid acute lymphocytic leukemia	NK cells are expanded *ex vivo* through co-culture with irradiated K562-mb15-41BBL and transduced with CD19-CAR	No preparative lymphodepletion regimen. Infusion of CAR-NK cells only	Assess safety and efficacy of modified NK cells, and determine highest tolerable dose of modified NK cells	-
None	Completed	N/A	NCT00187096	Acute myeloid leukemia	NK cell purification using CliniMACS system	Preparative lymphodepletion regimen followed by infusion of NK cells	Determine number of patients that exhibit grade 3 or 4 toxicities after NK cell infusion	19
None	Completed	1	NCT00586690	Lymphoma	NK cells were selected using a CD56 antibody and a Miltenyi Biotec system	Non-myeloablative stem cell transplant followed by infusion of NK cells	Determine toxicity of NK cell infusion	20
None	Completed	1	NCT01576692	Relapsed and refractory neuroblastoma in children	NK cell purification using CliniMACS system	Preparative lymphodepletion regimen with Hu14.18K322A (GD2 antibody). A second course of chemotherapy followed by NK cell infusion and Hu14.18K322A	Determine toxicities associated with administration of Hu14.18K322A with and without NK cell infusion in children	21
None	Completed	1	NCT00877110	Neuroblastoma; bone marrow, sympathetic nervous system	-	Preparative lymphodepletion regimen followed by infusion of escalating doses of NK cells and m3F8 (GD2 antibody)	Assess safety and feasibility of administering NK cell infusions with m3F8	22
None	Completed	2	NCT00526292	Leukemia, myelodysplastic syndrome	NK cell purification using CliniMACS system	Preparative lymphodepletion regimen followed by infusion of NK cells	Determine efficacy of NK cell infusion in patients who have undergone stem cell transplantation	23
None	Completed	2	NCT02395822	Acute Myelogenous Leukemia	NK cell purification using CliniMACS system and apheresis is depleted of CD3+ and CD19+ cells. NK cells are activated using IL-15	Preparative lymphodepletion regimen followed by infusion of NK cells and sub-cutaneous IL-15 administration	Measure disease progression and neutrophil counts 42 days after NK cell infusion	24
None	Completed	2	NCT01181258	Non-Hodgkin lymphoma, chronic lymphocytic leukemia	NK cells are expanded *ex vivo* and activated using IL-2	Preparative lymphodepletion regimen followed by infusion of NK cells and IL-2 administration	Evaluate patient response rate 2 months after NK cell infusion	25
None	Completed	2	NCT01390402	Leukemia, chronic myelogenous leukemia	NK cell purification using CliniMACS system and activated using IL-2	Infusion of NK cells after a preparative lymphodepletion regimen and before a peripheral blood stem cell transplantation	Determine response to stem cell transplantation after NK cell infusion and measure number of participants with complete remission 3 months after transplant	26
None	Active, not recruiting	1	NCT03081780	Refractory and relapsed acute myelogenous leukemia	NK cells are activated *ex vivo*	Preparative lymphodepletion regimen followed by single infusion of FT-NK100 and IL-2 administration	Determine maximal tolerable dosage of FATE-NK100	-
None	Active, not recruiting	1	NCT03319459	Solid tumors; HER2+ positive breast and gastric cancers, colorectal cancer, and EGFR1+ cancers	NK cells are activated and expanded *ex vivo*	No preparative lymphodepletion. Administration of only FT-NK100 for solid tumors; FT-NK100 with Trastuzumab (HER2+ antibody) for HER2+ tumors; FT-NK100 with Cetuximab (EGFR inhibitor) for EGFR1+ tumors	Observe incidences of dose limiting toxicity with FT-NK100	-
None	Recruiting	1	NCT03213964	Epithelial ovarian cancer, fallopian tube cancer, primary peritoneal cancer	NK cells are activated *ex vivo*	Preparative lymphodepletion regimen followed by intraperitoneal administration of escalating doses of NK cell product (FT-NK100) and IL-2 administration	Determine maximal tolerable dosage of FATE-NK100	-
None	Recruiting	1	NCT03209869	Neuroblastoma	NK cells are expanded and activated *ex vivo* through co-culture with K562-mbIL15-41BBL cells	Preparative lymphodepletion regimen followed by infusion of activated NK cells and administration of Hu14.18-IL2 (GD2 monoclonal antibody and IL-2 fused protein)	Assess safety of NK cells in combination with Hu14.18-IL2	-
None	Recruiting	1	NCT02890758	Myeloid malignancies, lymphomas, and sarcomas	NK cells are expanded *ex vivo*	Preparative lymphodepletion regimen followed by NK cell infusion. Some patients receive NK cell infusion with ALT803 (cytokine support), while others receive only NK cells.	Assess safety of NK cell infusion with ALT803, determine maximal tolerable dosage, and determining number of patients without graft vs host disease.	-
None	Recruiting	1	NCT03019666	B-cell malignancies, multiple myelomas, non-Hodgkin lymphomas	NK cells are expanded *ex vivo* using nicotinamide	Preparative lymphodepletion regimen followed by infusion of NK cells and IL-2 administration. Patients will also be given monoclonal antibodies for their respective conditions	Determine safety of escalating doses of NK cells and occurrences of grade 4 or higher adverse events and graft vs host disease	-

Genome Modification(s)	Status	Phase	Trial Number	Condition	Manufacturing Notes	Treatment Notes	Trial Goals	Ref.
None	Recruiting	2	NCT02100891	Ewing Sarcoma<br>Neuroblastoma<br>Rhabdomyosarcoma<br>Osteosarcoma<br>CNS Tumors	-	Preparative lymphodepletion regimen followed by bone marrow transplant and infusion of NK cells	Measure response rate and determine efficacy of NK cell infusion following a bone marrow transplant	-
None	Recruiting	1 and 2	NCT01787474	Recurrent and refractory adult acute myeloid leukemia	NK cells are expanded *ex vivo* using membrane-bound interleukin-21 (mbIL21)	Preparative lymphodepletion regimen followed by infusion of membrane bound IL-21 expanded NK cells	Determine safety, feasibility, and maximal tolerable dosage of NK cells	-
None	Recruiting	1 and 2	NCT01904136	Acute myeloid leukemia, myelodysplastic syndrome, or chronic myelogenous leukemia	NK cells are expanded *ex vivo*	Preparative lymphodepletion regimen followed by bone marrow transplant and NK cells	Determine safety, feasibility, and maximal tolerable dosage of NK cells in patients undergoing a stem cell transplant	-

Cord Blood NK Cells

Genome Modification(s)	Status	Phase	Trial Number	Condition	Manufacturing Notes	Treatment Notes	Trial Goals	Ref.
None	Active, not recruiting	1	NCT01619761	Hematological malignancies	NK cells are expanded *ex vivo*	Preparative lymphodepletion regimen followed by infusion of NK cells and umbilical cord blood transplant	Assess safety and feasibility of infusions of NK cells in patients with double cord blood transplantation	-
None	Recruiting	2	NCT01729091	Plasma cell leukemia and myeloma	-	Preparative lymphodepletion regimen followed by infusion of NK cells and autologous stem cell transplantation	Determine maximal tolerable dosage and efficacy of NK cells	-
None	Recruiting	2	NCT03019640	Refractory and recurrent B-cell non-Hodgkin's lymphoma	NK cells are expanded *ex vivo*	Preparative lymphodepletion regimen followed by stem cell transplant and infusion of NK cells	Determine treatment related mortality within 30 days of treatment	-
None	Recruiting	2	NCT02727803	Myelodysplastic syndrome, leukemia, lymphoma, multiple myeloma	-	Preparative lymphodepletion regimen followed by umbilical cord blood transplant and infusion of modified NK cells	Measure progression free survival	-
Anti-CD19 CAR (anti-CD19-CD28-zeta-2A-iCasp9-IL15)	Recruiting	1 and 2	NCT03056339	B-lymphoid malignancies, acute lymphocytic leukemia, chronic lymphocytic leukemia, non-Hodgkin lymphoma	NK cells are transduced with CD19-CAR	Preparative lymphodepletion regimen followed by infusion of CAR-NK cells	Assess safety and efficacy of CAR-NK cells, and determine optimal dose of CAR-NK cells	132

NK-92 Cells

Genome Modification(s)	Status	Phase	Trial Number	Condition	Manufacturing Notes	Treatment Notes	Trial Goals	Ref.
Engineered to produce endogenous IL-2 and express CD16 receptor (haNK)	Completed	1	NCT03027128	Solid tumors	-	No preparative lymphodepletion regimen. IV infusion of modified NK cells	Determine maximum tolerated dose of modified NK cells, and examine occurrence of dose limiting toxicities and adverse events	-
None	Completed	1	NCT00900809	Acute Myeloid Leukemia	-	No preparative lymphodepletion regimen. Infusion of escalating doses of NK cells given no adverse reactions after the first dose	Determine safety and maximum tolerable dose of NK cells (Neukoplast™)	27
None	Active, not recruiting	2	NCT02465957	Stage IIIB Merkel Cell Carcinoma<br>Stage IV Merkel Cell Carcinoma	NK cells are activated *ex vivo*	No preparative lymphodepletion regimen. Infusion of NK cells in combination with ALT-803 (cytokine support)	Determine progress free survival in patients 4 months after treatment (aNK, formerly Neukoplast™)	-
Engineered to produce endogenous IL-2 and express CD16 receptor (haNK)	Recruiting	2	NCT03853317	Merkel cell carcinoma	-	No preparative lymphodepletion regimen. IV infusion; Combination therapy of modified NK cells, N-803 (IL-15 superagonist), and Avelumab (PD-L1 antibody)	Evaluate efficacy of combination therapy and determine overall response rate	-
