# Supplementary material for: Genome engineering of induced pluripotent stem cells to manufacture natural killer cell therapies
Source: Stem Cell Res Ther. 2020 Jun 16;11:234. doi: 10.1186/s13287-020-01741-4 (PMC7298853; doi:10.1186/s13287-020-01741-4)
Supplement: Supplementary file 2 — Additional file 2. Summary of iPSC-NK Cell Therapies. The table summarizes the iPSC-NK cell therapies reviewed in the manuscript. [file 13287_2020_1741_MOESM2_ESM.pdf]

Supplementary Table 2. Summary of iPSC-NK Therapies (As of May 9, 2020)

Group	Product	Genome Modification(s)	Phase	Trial Number	Condition	Description	Ref.
Fate Therapeutics	FT500	None	1	NCT03841110 (Recruiting)	Solid tumors	Used in combination with checkpoint blockade therapy against solid tumors. Patients undergo preparative lymphodepletion regimen followed by infusion of FT500 and immune checkpoint inhibitor. Goals of the study are to measure patients with dose limiting toxicities	-
Fate Therapeutics	FT500	None	N/A	NCT04106167 (Recruiting)	Solid tumors	Measure long term survival and safety data from patients who participated in the parent FT500 trial (NCT03841110)	-
Fate Therapeutics	FT516	Engineered to include a high-affinity, non-cleavable CD16 (hnCD16) Fc receptor	1	NCT04023071 (Recruiting)	Hematologic Malignancies	Measure incidence of subjects with dose limiting toxicities. Administered in combination with monoclonal antibodies such as rituximab (anti-CD20) or Obinutuzumab (anti-CD20) or as a monotherapy.	-
Fate Therapeutics	FT538	CD38-less + hnCD16 + IL15/R	Preclinical	-	Multiple Myeloma	Engineered with knock-out of CD38 receptor and knock-in of high-affinity, non-cleavable CD16 receptor, and fused IL-15 receptor. To be used in combination with daratumumab (anti-CD38), monoclonal antibody	-
Fate Therapeutics	FT596	CAR19 + hnCD16 + IL15/R	Preclinical	-	Hematologic Malignancies	Engineered to include a NK cell-specific anti-CD19 CAR, high-affinity, non-cleavable CD16 receptor, and fused IL-15 receptor. Used in combination with rituximab (anti-CD20) monoclonal antibody	-
Kaufman	-	None	Preclinical	-	HIV/AIDS	iPSC-NK cells to target HIV infected CD4+ T cells	94
Kaufman	-	Engineered to include a recombinant receptor with CD4 extracellular domain and CD3z signaling chain	Preclinical	-	HIV/AIDS	iPSC-NK cells engineered with a recombinant CD4z receptor to target HIV infected CD4+ T cells	98
Kaufman	-	Anti-meso CAR with NKG2D, 2B4, CD3zeta signaling domains	Preclinical	-	Ovarian cancer xenograft model	Insertion of anti-meso CAR with NK specific signaling domains using *piggyback* transposons to increase antigen binding efficiency and anti-tumor activity	120
Kaufman	-	Site directed mutagenesis (S197P) in CD16a receptor	Preclinical	-	-	Site directed mutagenesis in the CD16a receptor (S197P) using a sleeping beauty transposon to prevent receptor cleavage and shedding upon NK activation (SKOV3 *in vitro* model)	121
Walcheck	-	Site directed mutagenesis (S197P) in CD16a receptor	Preclinical	-	-	Site directed mutagenesis in the CD16a receptor (S197P) using a *sleeping beauty* transposon to prevent receptor cleavage and shedding upon NK activation (K562 *in vitro* model)	122
Kaufman	-	Deletion of CISH gene using CRISPR	Preclinical	-	-	CISH gene (encoding for CIS regulatory element) was deleted using CRISPR/Cas9 to overcome negative regulation of IL-15 by CIS (K562 and MOLM-13 *in vitro* models)	123
Walcheck	-	Recombinant CD64/16A receptor	Preclinical	-	-	Insertion of a recombinant receptor with the extracellular domain of CD64, and the intracellular and transmembrane domain of CD16a using a *sleeping beauty* transposon to prevent CD16 receptor cleavage and shedding upon NK activation (SKOV3 *in vitro* model)	124
Kaufman	-	Engineered to include a high-affinity, non-cleavable CD16 (hnCD16) Fc receptor	Preclinical	-	-	Engineered to include a high-affinity, non-cleavable CD16 receptor to improve ADCC capabilities in hematologic malignancies and solid tumors	126
